# Supplementary material for: Natural Compounds Regulate Macrophage Polarization and Alleviate Inflammation Against ALI/ARDS
Source: Biomolecules. 2025 Jan 29;15(2):192. doi: 10.3390/biom15020192 (PMC11853067; doi:10.3390/biom15020192)

Figure S1. Chemical structures for important compounds from Table 1.

1. Rhein

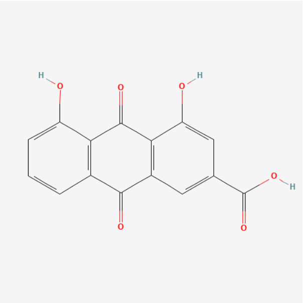

2. Loganin

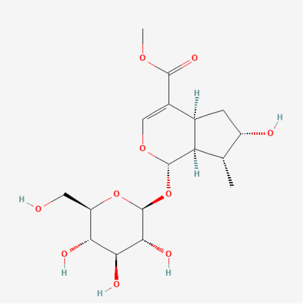

3. Salidroside

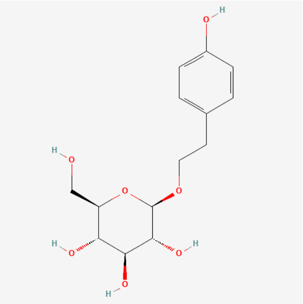

4. Ethyl ferulate

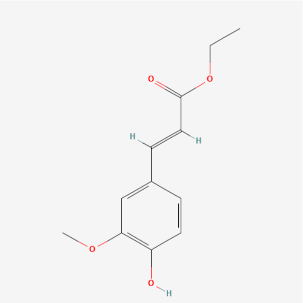

5. Resveratrol

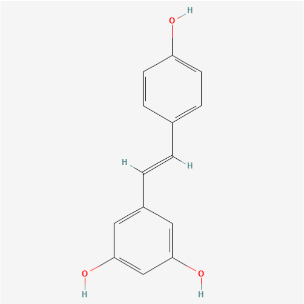

6. Allyl methyl trisulfide

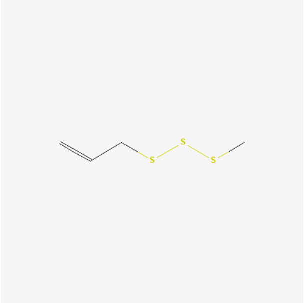

7. Cryptotanshinone

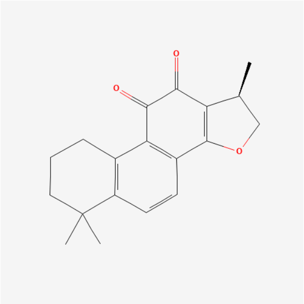

Supplement: Supplementary file 1 [file biomolecules-15-00192-s001.zip › biomolecules-3371653-supplementary.pdf]
